# Ethane groups modified DNA nanopores to prolong the dwell time on live cell membranes for transmembrane transport

**DOI:** 10.3389/fchem.2023.1148699

**Published:** 2023-02-28

**Authors:** Yuan Li, Xiaolei Chen, Cheng Lv, Yu Cheng

**Affiliations:** Translational Research Institute of Brain and Brain-Like Intelligence, Shanghai Fourth People’s Hospital, Tongji University School of Medicine, Shanghai, China

**Keywords:** ethane groups modified DNA nanopores, dwell time, live cell membranes, transmembrane transport, DNA nanotechnology

## Abstract

Transmembrane transport, mostly relying on biological channels, is crucial for the metabolic processes of live cells including sensing, signaling, cellular communicating and molecular transport. Artificial biomimetic channels offer excellent opportunities for studying the mechanisms of the metabolic processes of live cells and promote the applications of gene transfection, drug delivery, and regulations of cellular communications. DNA nanopores can be designed flexibly and operated easily while maintaining good biocompatibility, offering a good candidate for applications in basic research. However, because of the small size and good biocompatibility of DNA nanopores, it is still difficult to form stable channels on the plasma membrane of live cells by DNA nanopores. As a result, it significantly limits the applications of DNA nanopores *in vivo*. Thus, in this work, we have constructed ethane-phosphorothioate (PPT) groups modified DNA nanopores (E-DNA nanopores) to simulate biological channels for the transmembrane transport of small molecules. The E-DNA nanopores were found to be more hydrophobic and stable to anchor at the plasma membrane of live cells for a longer time window for subsequent transmembrane transport after the modification of ethane-PPT groups. The membrane-spanning E-DNA nanopores with a longer dwell time window could inspire the design of new DNA nanostructures and expand their biological applications including biosensing and sequencing, construction of artificial cells and regulation of transmembrane transport.

## 1 Introduction

The cells’ behavior to maintain metabolic activities including material transport, information exchange and sensing communication depends on transmembrane transport. Artificially regulating the transport of various small molecules such as inorganic ions, biomolecules, and drugs, is of great significance for gene editing (Wilbie et al., 2019), drug delivery (Yan et al., 2014), disease treatment (Naldini, 2015; Stewart et al., 2018) and other fields. Existing approaches that use viruses (Dunbar et al., 2018; Raguram et al., 2022), external fields including light (Lyu et al., 2016; Wayteck et al., 2017), sound (Crowley et al., 2016; Belling et al., 2020; Chowdhury et al., 2020) and electricity (Fletcher et al., 2010; Xie et al., 2013; Kotnik et al., 2019) and harsh chemical reagents (Quebatte et al., 2014; Stewart et al., 2016) are complex, costly and induce cellular stress and toxicity. Constructing biomimetic channels on live cells provides excellent prospects for convenient, safe and effective regulation of transmembrane transport. With the development of DNA nanotechnology (Gopfrich et al., 2016; Gale et al., 2017; Hu et al., 2019), DNA nanopores have demonstrated significant application potential in the field of cell biology in recent years (Lv et al., 2020; Lanphere et al., 2021a; Arulkumaran et al., 2021). As a new material, DNA nanopores can be designed flexibly and operated easily while maintaining low cell toxicity and good biocompatibility (Langecker et al., 2012). Considering their unique self-recognition and sequence programmability, predictable DNA nanopores could be assembled to form a channel structure that provides a pathway for the transmembrane transport of small molecules (Howorka, 2017). Moreover, DNA nanopores can be chemically functionalized via the incorporation of appropriate DNA bioconjugates, which can extend the applications of DNA nanopores in the field of biology and promotes the combination of DNA nanotechnology and biochemistry (Bae et al., 2019; Madsen and Gothelf, 2019). However, the insertion and stable anchoring of DNA nanopores in living cells are difficult because of the strong contradictory energy interactions between the hydrophilicity and electronegativity of the phosphate group of DNA nanopores and the hydrophobic environment of membranes (Maingi et al., 2017). Although DNA nanopores could be chemically modified to carry hydrophobic, neutral-charged groups or hydrophobic lipid anchors (Burns et al., 2014; Burns et al., 2016) to overcome the energetic barrier to bilayer insertion, it is still a big challenge to prolong the dwell time of DNA nanopores on the plasma membrane of live cells and to form stable channels (Whitehouse et al., 2019).

**FIGURE 1:**
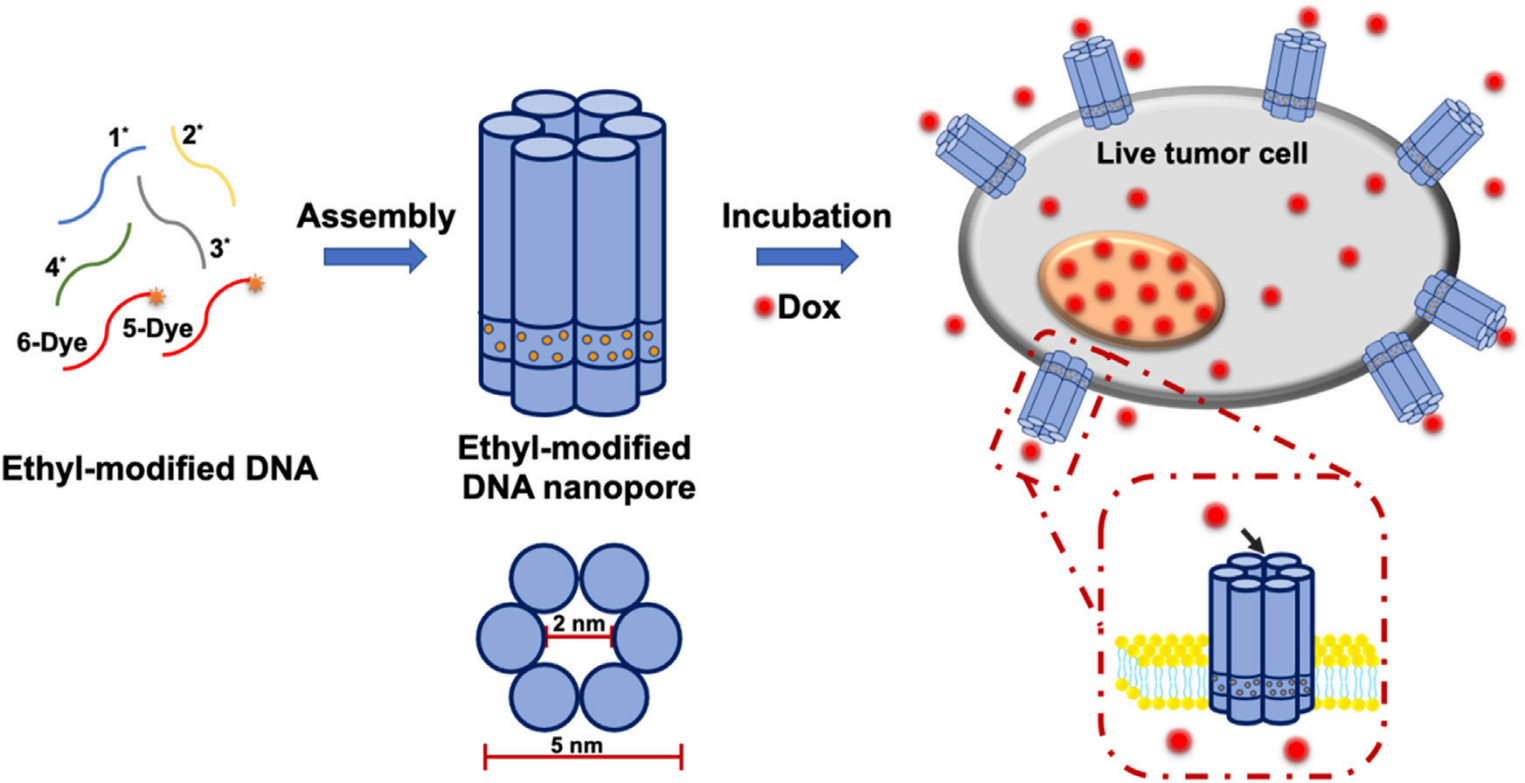
Illustration of the construction of E-DNA nanopores and the insertion of E-DNA nanopores in the plasma membrane of live cells.

Thus, in this work, DNA-based nanopores with long membrane dwell time were constructed through chemical modifications. Moreover, they were inserted in the plasma membrane of live cells for small molecules transmembrane transport. As shown in. Supplementary Table S1, ssDNA 1*, 2*, three* and four* were modified with hydrophobic phosphorothioate (PPT) groups. Meanwhile, ssDNA five and six were labeled with TAMRA or Cy5 fluorophores for imaging. In order to neutralize the electronegativity of DNA nanopores for cell membrane insertion and anchoring, ssDNA 1*, 2*, three* and four* were modified with positively charged ethane and then purified by HPLC (Supplementary Figure S1). Subsequently, E-DNA nanopores were acquired by assembling ethane-PPT-modified ssDNA 1*, 2*, 3*, four* and fluorophores labeled ssDNA five and 6. As shown in Figure 1, E-DNA nanopores consisted of six DNA duplexes with a theoretical inner diameter of 2 nm, an outer diameter of 5 nm, and a height of 14 nm. At the bottom of the pore wall, 72 ethane-PPT groups formed a hydrophobic charge neutralization band with a height of 2 nm, which promoted the membrane insertion of E-DNA nanopores by reducing the energy barrier between the hydrophilic electronegativity of E-DNA nanopores and the hydrophobic environment of the membrane. This modification also allowed E-DNA nanopores to present an energy barrier that prevented their escaping from the membrane (Schinkel and Jonker, 2003; Maingi et al., 2017). Therefore, the ethane-PPT groups modified DNA nanopores may prolong the dwell time of DNA nanopores on the plasma membrane of live cells, open an efficient window for small molecules transmembrane transport, and broaden the application range of DNA nanotechnology.

## 2 Materials and methods

### 2.1 Materials

DNA oligonucleotides were synthesized by Sangon Biotech Co., Ltd. (Shanghai, China) according to the reference (Burns et al., 2014). Doxorubicin hydrochloride (Dox), 4 S Red Plus Nucleic Acid Stain and 10 × TAE buffer were purchased from Sangon Biotech Co., Ltd (Shanghai, China). Fetal bovine serum (FBS), PMI-1640 medium, Penicillin, and Streptomycin was obtained from Gibco. CellMaskTM deep red plasma membrane stain was purchased from Life Technologies. Michigan Cancer Foundation (MCF-7) cell line was obtained from American Type Culture Collection (ATCC, United States). Dox-resistant MCF-7 (MCF-7/Adr) cell line was bought from Shanghai Gefan Biotechnology Co., Ltd. and 1 Kb DNA ladder were purchased from Yeasen Biotech Co., Ltd. Propidium iodide (PI) were purchased from Beyotime (Shanghai, China) and Calcein-AM was bought from Invitrogen (Shanghai, China).

### 2.2 Construction of E-DNA nanopores

According to the reference (Burns et al., 2013), PPT groups modified DNA oligonucleotides (1^*^, 2^*^, three^*^ and 4^*^, Supplementary Table S1) were dissolved in the mixture of 90% DMF and 10% 30 mM Tris-HCl (pH 8.0). One-fifth by volume of iodoethane was then added to the solution. After heating at 65°C for 1.5 h, the mixture was freeze-dried overnight. To obtain ethane-PPT groups modified DNA oligonucleotides (E-1^*^, E-2^*^, E-3^*^, and E-4^*^), the products were purified by HPLC to remove abundant iodoethane and unmodified DNA oligonucleotides. Subsequently, 1 μM of each of the four modified DNA oligonucleotides (E-1*, E-2*, E-3*, and E-4*) and the other two DNA oligonucleotides (5-Dye and 6-Dye) were mixed in 1 × TAE containing 12.5 mM MgCl_2_. To synthesize E-DNA nanopores, the mixture was heated up to 95°C for 5 min and then cooled down to 16°C at a rate of 0.5°C per min in a PCR amplifier. The samples were then purified by ultrafiltration.

### 2.3 Characterization of E-DNA nanopores

The assembled E-DNA nanopores were analyzed by 1% agarose gel electrophoresis while using 1 × TAE buffer. The agarose gel was pre-stained by 4S Red Plus Nucleic Acid Stain. For gel loading, a solution of 1 μL E-DNA nanopores, 1 μL 6 × loading buffer was mixed with 4 μL 1 × TAE buffer. The gel was run for 60 min at 70 V at room temperature. The bands were then visualized by UV illumination (Amersham imager 680). Transmission electron microscope (TEM) and atomic force microscope (AFM) were used to determine the morphology and detailed dimension of E-DNA nanopores. An appropriate amount of E-DNA nanopores were dropped on carbon-coated copper grids and incubated for 10 min before being drained with filter paper. 10 μL 2% sodium phosphotungstate was immediately added to the copper grids and incubated for 5 min for negative staining. After drying with filter paper, the samples were rinsed three times with ultra-pure water. The TEM images were captured by transmission electron microscope (HT7700, Hitachi). Initially, 5 μL E-DNA nanopores were mixed with 40 μL 1 × TAE buffer containing 10 mM MgCl_2_ and 30 mM NiCl_2_. An appropriate amount of the mixture was spread on the surface of freshly treated mica and incubated for about 2 min. The AFM images were obtained using a Multimode 8 system (Bruker Corp).

### 2.4 Working condition optimization of E-DNA nanopores on live cells

MCF-7 cells were pre-planted in 35 mm glass-bottom confocal dishes at a concentration of 50,000 cells per dish. Different concentrations of E-DNA nanopores were then added to the dishes for co-incubation at 4°C. E-DNA nanopores were labeled with Cy5 fluorophores. The nucleus of MCF-7 cells was stained by Hoechst 33,342 and the plasma membrane was labeled by CellMask deep red. The prepared samples were examined using confocal microscopy (Leica TCS SP8) and flow cytometry (CytoFLEX LX). The confocal images were analyzed by Imaging-Pro-Plus software (Media Cybernetics). The flow cytometry results were analyzed by Flowjo software (Vienna, Austria).

### 2.5 Characterization of E-DNA nanopores for transmembrane transport

The MCF-7 cells planted in 35 mm glass-bottom confocal dishes or 24-well plates were left overnight for culture. Subsequently, 100 nM E-DNA nanopores were added into the dishes/wells and incubated at 4°C for 40 min. The cells were then incubated with a mixture of Calcein-AM and PI at 37°C for 40 min. The prepared samples were examined by confocal microscopy and flow cytometry. The confocal images were analyzed by Imaging-Pro-Plus software. The flow cytometry results were analyzed by Flowjo software.

The MCF-7 and MCF-7/Adr cells pre-planted in 35 mm glass-bottomed confocal dishes were first incubated with 100 nM E-DNA nanopores at 4°C for 40 min. The excess nanopores were removed by three times wash in 1 × PBS. MCF-7 cells with E-DNA nanopores inserted were then co-incubated with Dox at 37°C for 20 or 40 min, while MCF-7/Adr cells with E-DNA nanopores inserted were co-incubated with 0.15 mg/ml Dox at 37°C for 2 h. To measure the drug delivery efficiency of E-DNA nanopores, confocal microscopy was used to detect the fluorescence signals. The confocal images were analyzed by Imaging-Pro-Plus software.

The MCF-7 cells pre-planted in 96-well plates and E-DNA nanopores were inserted into the cells under the same conditions. The excess nanopores were removed by three times wash in 1xPBS. MCF-7 cells with E-DNA nanopores inserted were then co-incubated with different concentration of Dox at 37°C for 40 min. To remove the abundant Dox, the prepared samples were washed three times in 1×PBS and then incubated at 37°C for 48 h. The cell viability was detected by CCK8-kit. The MCF-7 and MCF-7/Adr cells pre-planted in 96-well plates and E-DNA nanopores were inserted into the cells under the same conditions. The excess nanopores were removed by three times wash in 1xPBS. MCF-7 and MCF-7/Adr cells were then co-incubated with 0.15 mg/ml Dox at 37°C for 40 min and 2 h respectively. To remove the abundant Dox, the prepared samples were washed three times in 1 × PBS and then incubated at 37°C for different time. The cell viability was detected by CCK8-kit.

### 2.6 Live cell fluorescence imaging and image analyses

The Hoechst 33,342, Calcein-AM, PI/TAMRA/Dox, and CellMask deep red/Cy5 were excited by 405, 488, 561 and 640 nm lasers, respectively. The emission signals were collected according to the emission spectra of organic dyes and recorded by sCMOS. The confocal images were analyzed by Imaging-Pro-Plus software.

## 3 Results and discussion

### 3.1 Characterization of E-DNA nanopores

**FIGURE 2:**
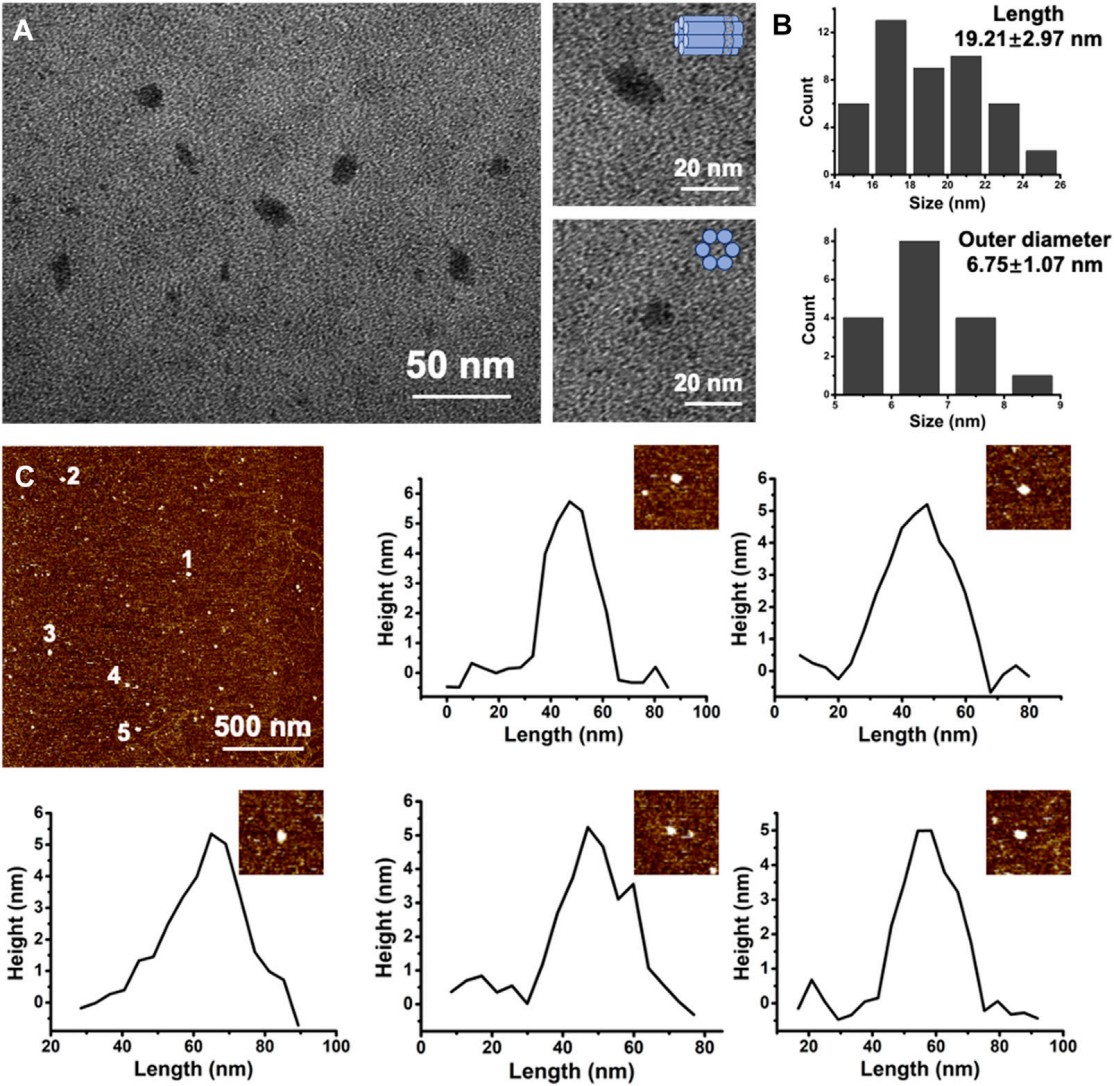
**(A)** Representative TEM image of E-DNA nanopores. Scale bar: 50 nm. High-magnified TEM images of single E-DNA nanopores of different positions. Scale bar: 20 nm. **(B)** Statistic results of the length and outer diameter of E-DNA nanopores (*n* = 46 for length and *n* = 17 for outer diameter). **(C)** Representative AFM image of E-DNA nanopores and the corresponding height analysis. Scale bar: 500 nm.

To ensure the formation of E-DNA nanopores, the chemical modification of ethane-PPT groups on DNA nanopores was determined by HPLC. The HPLC results showed that most of the E-DNA nanopores were eluted later than PPT groups modified DNA nanopores. It indicated that the chemical modification of the ethyl groups was successful (Supplementary Figure S1). Native agarose gel electrophoresis results showed that six DNA oligonucleotides were assembled into a uniform structure (Supplementary Figure S2, line 1). The band of E-DNA nanopores appeared at around 250 bp, which was similar to the theory. Subsequently, the morphology and the detailed dimension of DNA nanopores were measured by TEM and AFM analysis. The morphology of E-DNA nanopores was determined to be cylindrical based on TEM images (Figure 2A). Based on the statistical results of TEM images, the size of E-DNA nanopores was determined to be relatively uniform with an average length of 19.21 ± 2.97 nm (Figure 2B, *n* = 46) and an outer diameter of 6.75 ± 1.07 nm (Figure 2B, *n* = 17). They were consistent with the theoretical dimensions. According to the statistical results of AFM images, the height of E-DNA nanopores was found to be about 4.98 ± 0.83 nm. It was consistent with the theoretical dimension (5 nm) after correcting for tip deconvolution (Figure 2C and Supplementary Figure S2, *n* = 23).

### 3.2 The dwell time of E-DNA nanopores on live cells

**FIGURE 3:**
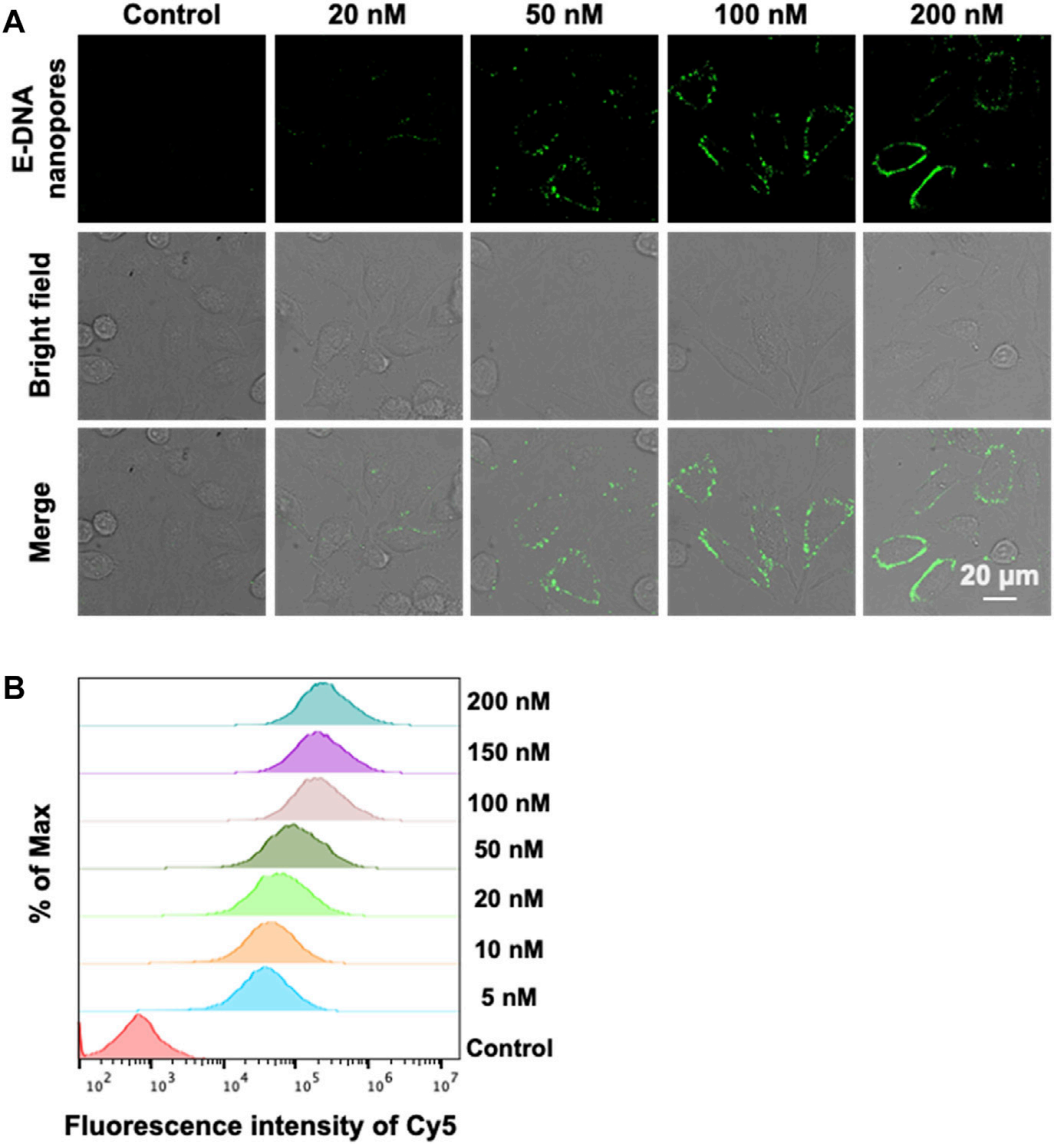
**(A)** Confocal images of MCF-7 cells incubated with different concentrations of E-DNA nanopores. Scale bar: 20 μm. **(B)** Flow cytometry analysis of MCF-7 cells incubated with different concentrations of E-DNA nanopores.

The MCF-7 cell, a common model of breast cancer, was used in this study to demonstrate that E-DNA nanopores could be effectively inserted into the plasma membrane of live cells. The nanopores were modified with Cy5 to understand their distribution on the membrane surface. The concentration of E-DNA nanopores incubated with cells was first optimized. Different concentrations of E-DNA nanopores were incubated with MCF-7 cells. The fluorescence signals of Cy5 labeled E-DNA nanopores were detected by confocal microscopy and flow cytometry. Confocal images showed that the fluorescence signal of E-DNA nanopores could be detected on the plasma membrane of all concentration groups (Figure 3A), indicating an effective insertion on the cell membrane of E-DNA nanopores. The fluorescence intensity increased with the concentration of E-DNA nanopores and showed a relatively uniform and bright fluorescence signal distribution when the concentration of E-DNA nanopores reached 100 nM. However, when the concentration of E-DNA nanopores reached 200 nM, several large flaky or linear fluorescent spots became apparent in the image (Figure 3A). It suggested that the self-aggregation was caused by the saturated concentration of E-DNA nanopores. Furthermore, the flow cytometry results showed that the fluorescence intensity reached its maximum when the E-DNA nanopores concentration reached 100 nM (Figure 3B). It indicated that 100 nM was the saturation concentration, having similar results as confocal images. Therefore, 100 nM of E-DNA nanopores incubated with MCF-7 cells were chosen as our working concentration.

**FIGURE 4:**
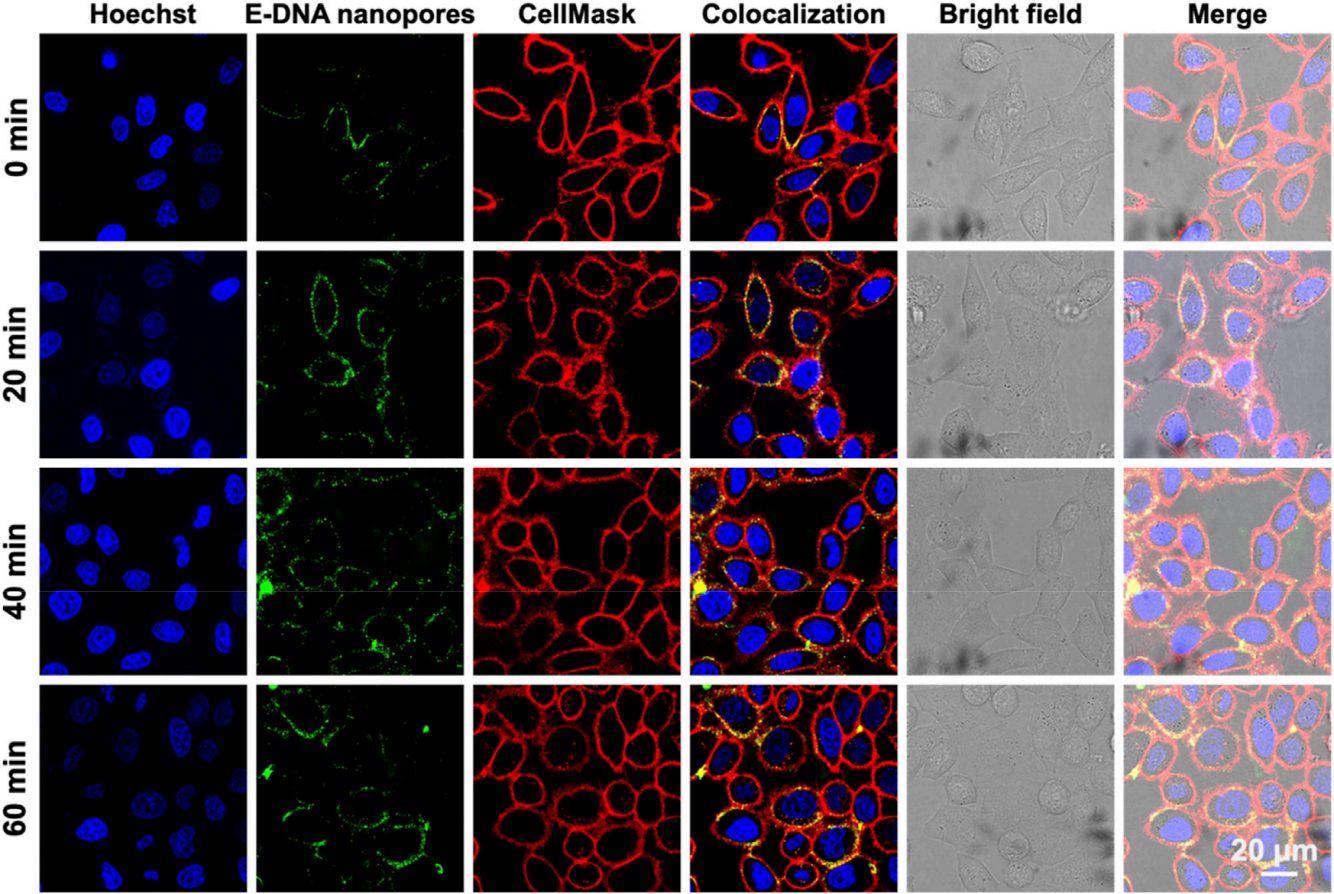
Confocal images of MCF-7 cells incubated with E-DNA nanopores at different times. Scale bar: 20 μm.

Subsequently, the dwell time of E-DNA nanopores at the plasma membrane of live cells was measured. The 100 nM E-DNA nanopores were incubated with MCF-7 cells for different amounts of time. The fluorescence signals were characterized by confocal microscopy. To determine the location of the cell membrane, the membrane dye CellMask deep red was used to label the plasma membrane of MCF-7 cells. Confocal images showed that under different incubation times, most of the green fluorescence signals (pseudo color, TAMRA labeled E-DNA nanopores) were co-located along with the red fluorescence signals (plasma membrane) (Figure 4). In addition, with the extension of incubation times, the intensity and distribution of green fluorescence signals on the cell membrane remained stable. Even with the incubation time of 60 min, E-DNA nanopores were still firmly anchored on the cell membrane, neither falling off the membrane nor being internalized by cells. It indicated that 20–60 min could be used as working incubation time and E-DNA nanopores could stay at the plasma membrane of live cells for at least 60 min.

To further demonstrate the advantage of long dwell time of E-DNA nanopores on the cell membrane, PPT group-modified DNA nanopores (DNA nanopores) were constructed and labeled by Cy5 for further characterization. The dwell times of the E-DNA nanopores and DNA nanopores were then compared. As shown in Supplementary Figure S3, after incubation at 37°C for 20 min, relatively uniform fluorescence signals could be generated on the surface of the cell membrane. It indicated that DNA nanopores also had rapid and effective membrane insertion properties. However, after incubation at 37°C for 40 min, partial fluorescence signals of DNA nanopores were found to be distributed in the cytoplasm, which indicated that DNA nanopores started to internalize into MCF-7 cells. For E-DNA nanopores, the fluorescence signals were consistently distributed around the plasma membrane of live cells at all periods even after incubation at 37°C for 60 min. Therefore, with the modification of ethane-PPT groups, E-DNA nanopores could stay at the plasma membrane of live cells for a longer time (≥60 min), which could open a long-term channel for small molecules to enter cells. To further demonstrate the biosafety of DNA nanopores, a CCK-8 assay was performed. No significant toxicity of E-DNA nanopores was found (Supplementary Figure S4), indicating that modification of ethane could prolong the dwell time of E-DNA nanopores without increasing their biotoxicity. Since E-DNA nanopores are open and lack directional control over material transportation, the possible leakage of small molecules from the cell inside has been discussed. As the hydrophilicity, electronegativity and size of E-DNA nanopores allow them to selectively transport certain substance, only hydrophilic and smaller than 2 nm molecules can be transported and limit the leakage significantly (Zhu et al., 2019; Lanphere et al., 2021b). The cell viability of cells incubated with E-DNA nanopores remained similar to the control group (cells only). It indicated that the small molecules leakage from our constructed E-DNA nanopores was too little to influence the viability of cells. As per our understanding, the possible leakage of small molecules did not affect the further biological application of E-DNA nanopores.

### 3.3 Transmembrane transport of small molecules via E-DNA nanopores inserted on live cells

Subsequently, the passable channel by the E-DNA nanopores was constructed on the plasma membrane of live cells to carry forward the applications of E-DNA nanopores on transmembrane transport. Propidium iodide (PI), a small red-fluorescent molecule, was used as a probe to verify the transmembrane transport of small molecules via E-DNA nanopores because it could not traverse live cells with the intact plasma membrane. As shown in Supplementary Figure S5, only the fluorescence signals of calcein-AM were observed in normal MCF-7 cells while the fluorescence signals of PI were absent. It could be related to the blocking of intact membranes in live cells. However, after the insertion of E-DNA nanopores, both green and red fluorescence signals appeared in the cells, indicating that E-DNA nanopores mediated the transport of PI to MCF-7 live cells. Besides, the flow cytometry results showed a significantly enhanced PI fluorescence intensity in MCF-7 cells inserted with E-DNA nanopores (Supplementary Figure S5), which was similar to the confocal imaging results. Taken together, the small molecule PI, which could not traverse the membrane of live cells, can enter live MCF-7 cells by inserting E-DNA nanopores. It suggested that E-DNA nanopores possess the ability to efficiently form artificial channels on the plasma membrane of live cells, which can be used as a transmembrane transport pathway for small molecules.

**FIGURE 5:**
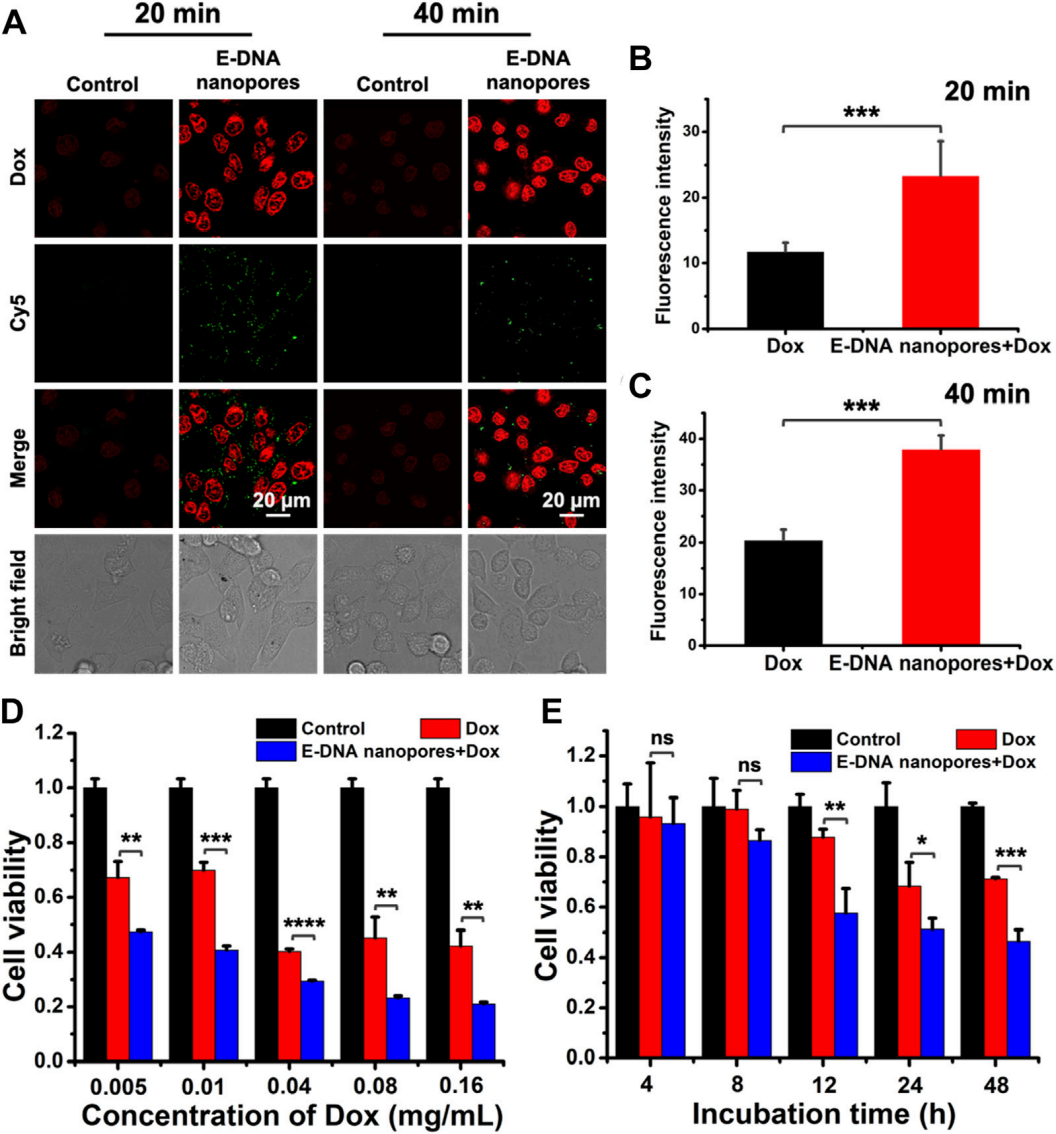
**(A)** Confocal images of MCF-7 cells inserted with DNA nanopores and incubated with Dox at 37°C for a different time. Scale bar: 20 μm. **(B–C)** The statistic of average fluorescence intensity of Dox in each cell with different incubation times (*n* = 40). *** indicates *p* < 0.001. **(D)** Cell viability of MCF-7 cells incubated with different concentration of Dox for 40 min and then incubated at 37 °C for 48 h without extra Dox (*n* = 3). ** indicates *p* < 0.01. *** indicates *p* < 0.001. **** indicates *p* < 0.0001. **(E)** Cell viability of MCF-7 cells incubated with 0.15 mg/ml Dox for 40 min and then incubated at 37°C for different time without extra Dox (*n* = 3). ns indicates no significant difference. * indicates *p* < 0.05. ** indicates *p* < 0.01. *** indicates *p* < 0.001.

Furthermore, E-DNA nanopores were established on the surface of live tumor cell membrane for the transmembrane transport of the small molecule drug Dox. It was an attempt to apply E-DNA nanopores to cancer therapy. The MCF-7 cells with (experimental group) or without (control group) E-DNA nanopores were incubated with Dox at 37 °C for a different time and captured by a confocal microscope. The fluorescence signals of Cy5 labeled E-DNA nanopores were evident on the live MCF-7 cells membrane while the control group showed no green fluorescence signals around MCF-7 cells. Meanwhile, Dox signals in MCF-7 cells inserted with E-DNA nanopores were much brighter than those in the control group (Figure 5A). The statistical results showed that the fluorescence intensity of Dox was significantly different (*p* < 0.001) between the two groups, indicating that E-DNA nanopores could effectively mediate the transport of Dox to MCF-7 cells (Figures 5B, C). Additionally, the fluorescence intensity of Dox inside MCF-7 cells inserted with E-DNA nanopores increased with the incubation time. It indicated that a longer dwell time of E-DNA nanopores could enhance drug delivery efficiency. Moreover, the cell viability of MCF-7 cells inserted with or without E-DNA nanopores incubated with different concentrations of Dox was measured. The results showed that the cell viability of MCF-7 cells inserted with E-DNA nanopores and incubated with Dox was much lower than other groups (Figure 5D). Even with a very low concentration of Dox (0.005 mg/ml), the cell viability of MCF-7 cells inserted with E-DNA nanopores decreased to about 50%, which was significantly different (*p* < 0.01) than the only Dox group with 75% cell viability. These results further indicated that E-DNA nanopores could lead to higher Dox entry into cells at consistent dosing concentrations. Furthermore, the cell viability of MCF-7 cells inserted with or without E-DNA nanopores incubated with Dox for different time was also measured. The results showed that the cell viability of the MCF-7 cells with the E-DNA nanopores insertion group was lowest at different incubation times with Dox (Figure 5E), which was consistent with previous results. In addition, cell viability decreased with time for all groups, which might be related to the gradual toxic effects of intracellular Dox over time. The cell viability of the experimental group (MCF-7 cells inserted with E-DNA nanopores and incubated with Dox) dropped more obviously within a short time. After co-incubation with Dox for 40 min, the viability of normal tumor cells decreased significantly after 12 h in normal culture medium. Whereas, tumor cells inserted with E-DNA nanopores showed a significant decrease in cell viability after 4 h in normal medium under the same conditions. These results suggested that E-DNA nanopores provided a direct transmembrane transport pathway that allowed Dox to enter the cell quickly and exert cytotoxicity. Notably, there may be several reasons for the discrepancy of the similar experimental condition shown in Figure 5D (MCF-7 cells inserted with E-DNA nanopore and then incubated with 0.16 mg/ml Dox for extra 48 h without Dox) and 5 E (MCF-7 cells inserted with E-DNA nanopore and then incubated with 0.15 mg/ml Dox for extra 48 h without Dox). First, because of the complex characters of biological system, the state of cells has a great influence on the results of different experimental batches. As we can see from Figure 5E, the group of Dox, the cell viability decreased to 70% after incubating with 0.15 mg/ml Dox for 48 h (without Dox). While the cell viability of the same group shown in Figure 5D was around 40% by incubating with 0.16 mg/ml Dox for 48 h (without Dox), indicating that the cells may be more active to uptake small molecules from the surroundings. In our experiments, even in the experimental group (E-DNA nanopores + Dox), the internalized Dox included the free diffusion from surroundings and transport through E-DNA nanopores. Therefore, according to the various results shown in the Dox only group in Figures 5D, E. We think that the state of cells in the two different experiments were quite different and may be the main reason for the batch variances. Second, the concentration of cells planted in 96-well plates also contributes significantly to the results of different experimental batches. In detail, the initial concentration of cells determines the average concentration of E-DNA and Dox on each cell and thus decreases both the transport efficiency of E-DNA nanopores and the pharmaceutical effect of Dox. Taken together, we believe that the discrepancy was caused by the batch variance.

**FIGURE 6:**
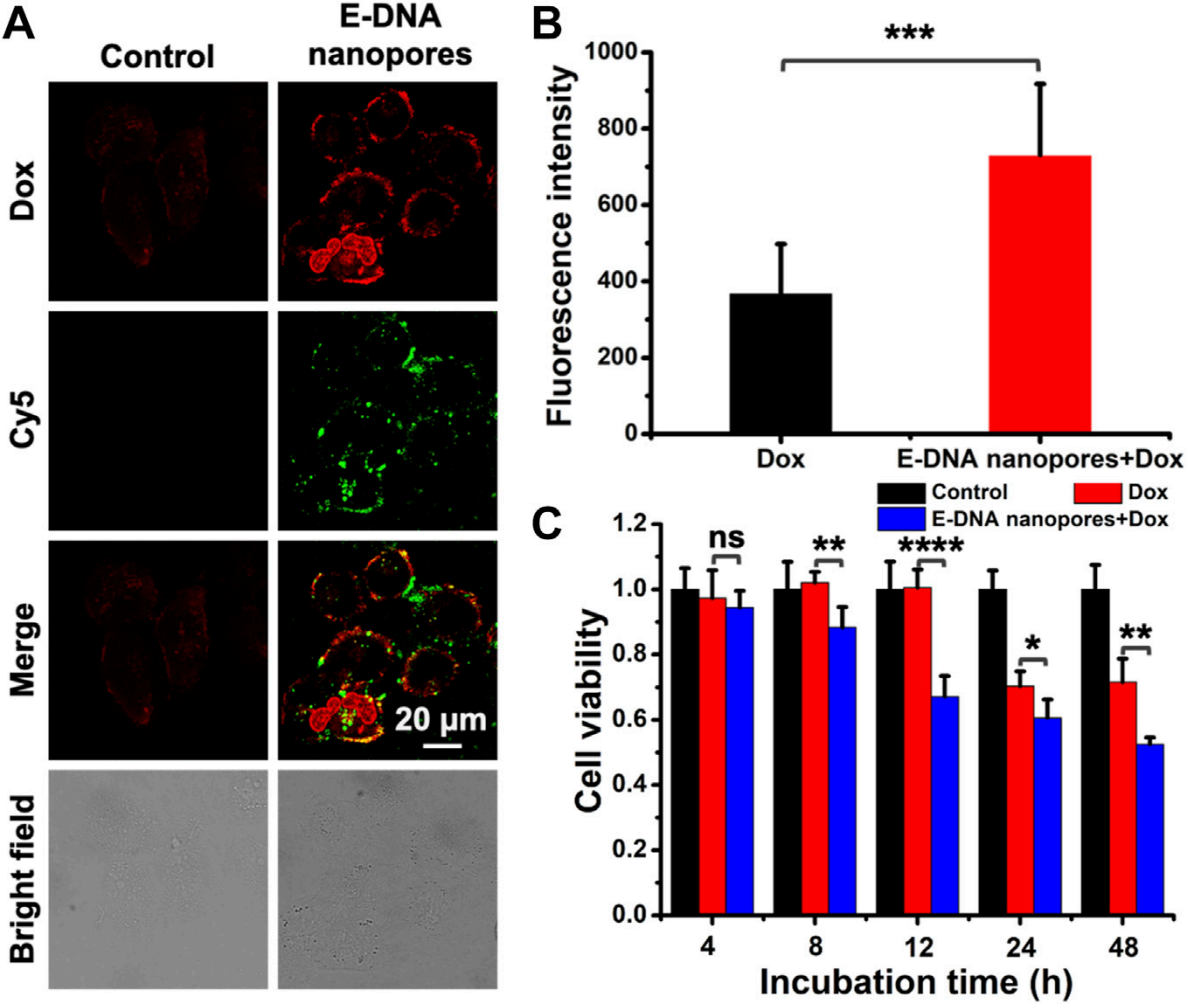
**(A)** Confocal images of MCF-7/Adr cells inserted with DNA nanopores and incubated with Dox at 37°C for 2 h. Scale bar: 20 μm. **(B)** The statistic of average fluorescence intensity of Dox in each MCF-7/Adr cell (*n* = 20). *** indicates *p* < 0.001. **(C)** Cell viability of MCF-7 cells incubated with 0.15 mg/ml Dox at 37°C for 2 h and then incubated at 37°C for different time without extra Dox (*n* = 5). ns indicates no significant difference. * indicates *p* < 0.05. ** indicates *p* < 0.01. **** indicates *p* < 0.0001.

Considering the fact that tumor cells could be resistant to the drugs during treatment, drilling a passive channel on the plasma membrane of drug-resistant cells may provide the opportunity for drug molecules to enter drug-resistant cells effectively. Therefore, in order to demonstrate the effect of E-DNA nanopores on drug-resistant cell lines, Dox-resistant MCF-7 (MCF-7/Adr) cell line was chosen as a model cell line. Similarly, the green fluorescence signals indicated that E-DNA nanopores could be effectively inserted into the plasma membrane of MCF-7/Adr cells membrane (Figure 6A). In addition, due to the drug resistance of MCF-7/Adr cells, almost no Dox fluorescence signals were observed in normal MCF-7/Adr cells while obvious Dox fluorescence signals appeared inside MCF-7/Adr cells inserted with E-DNA nanopores (Figure 6A). According to the statistical data of Dox fluorescence intensity, significant differences (*p* < 0.001) were found between the two groups. It indicated that E-DNA nanopores could facilitate the entry of Dox into MCF-7/Adr cells which were difficult for chemotherapy drugs to enter (Figure 6B). The cell viability of MCF-7/Adr cells inserted with or without E-DNA nanopores incubated with Dox was also measured by CCK-8 assay. The cell viability of MCF-7/Adr cells inserted with E-DNA nanopores was much lower than other groups and the differences among them were more significant (Figure 6C). Since Dox can be actively pumped out of the cells through protein channels on the MCF-7/Adr cells membrane, the cell viability only decreased to 70% at 48 h. The cell viability of MCF-7/Adr cells inserted with E-DNA nanopores decreased after 8 h incubation with Dox. It even decreased to 50% at 48 h, which was significantly different (*p* < 0.01) from the only Dox group. These results further confirmed that E-DNA nanopores could open transmembrane transport channels on drug-resistant tumor cells and enhance the entrance of Dox to drug-resistant tumor cells efficiently while exhibiting a more lethal effect.

## 4 Conclusion

In summary, we have established bio-inspired membrane-spanning E-DNA nanopores with a longer dwell time on the plasma membrane of live cells. Considering the hydrophobic, charge-neutral ethane-PPT groups, the E-DNA nanopores could efficiently insert into the plasma membrane of live cells and stay for more than 1 h at 37°C, which was longer than most DNA nanopores. In addition, taking advantage of the long dwell time and good biocompatibility, the E-DNA nanopores exhibited no significant cytotoxicity to live cells, offering opportunities for their biological application. By utilizing these E-DNA nanopore-based memetic channels, we have demonstrated the effective transmembrane transport of small molecules in live cells such as drug delivery on tumor cells and drug-resistant tumor cells. Thus, our work provides a new horizon for combining DNA nanotechnology and biology and offers possibilities of DNA nanostructures for research of biosensing, catalysis, drug delivery and nanofluidics on live cells. In the future, we hope our E-DNA nanopores would further inspire more studies on biomimetic channels on live cell membranes, such as the modification of targeted molecules (antibodies, aptamers and small molecules) on DNA nanopores and the combination of some anti-nuclease technologies (covalent cross-linking and surface coating) (Kizer et al., 2019; Chandrasekaran, 2021). Herein, the biological and clinical applications of DNA nanopores will be greatly expanded.

## Data Availability

The original contributions presented in the study are included in the article/Supplementary Material, further inquiries can be directed to the corresponding authors.
