# Supplementary material for: Ethane groups modified DNA nanopores to prolong the dwell time on live cell membranes for transmembrane transport
Source: Front Chem. 2023 Feb 28;11:1148699. doi: 10.3389/fchem.2023.1148699 (PMC10011181; doi:10.3389/fchem.2023.1148699)
Supplement: Supplementary file 1 [file Presentation1.pdf]

Supplementary Material

1 Supplementary Materials and Methods

1.1. Cytotoxicity of E-DNA nanopores

MCF-7 cells were planted in 96-well plates in advance. Then, the cells were incubated with different concentrations of E-DNA nanopores for 2 h or incubated with 100 nM E-DNA nanopores for different time. The cell viability was detected by CCK8-kit.

1.2. Dwell time of E-DNA nanopores on live cells

MCF-7 cells were pre-planted in 35 mm glass-bottom confocal dishes at a concentration of 50,000 cells per dish. DNA nanopores or E-DNA nanopores were added to the dishes for co-incubation at 4°C for 40 min, and then continued incubated at 37°C for different time. The prepared samples were examined by confocal microscopy (Leica TCS SP8). TAMRA were excited by 561 nm laser, and the emission signals were collected according to the emission spectra of organic dyes and recorded by sCMOS. The confocal images were analyzed by Imaging-Pro-Plus software (Media Cybernetics).

2 Supplementary Figures and Tables

2.1 Supplementary Table

Supplementary Table 1. Sequences of DNA oligonucleotides for assembling the DNA nanopore.

#	Sequences（5'→3'）
**1***	ACA*G*G*A*T*T*TTCGCCTGCTGGGGCAAACCAGCGTGGACCGCTTTTTTGGCTAT TCTTTTGATTTATAAGGGATTTTGCCGA*T*T*T*C*G*GAA
**2***	CAACTCTCTCAGGGCCAGGCGGTGAAGGGCAATC*A*G*C*T*G*TTGTTTTCAA*C* A*G*C*A*T*C*C*TGTTTC*C*G*A*A*A*TCGGCATTAAAG*A*C*CAGCTG
**3***	TCT*C*A*C*T*G*GTGAAAAGAAAAACCACCCTGGCGCCCAATACGCTTTTTCCCCG CGCGTTGGCCGATTCATTAATGCAGCTG*G*C*A*C*G*ACA
**4***	GGCGAA*A*T*GATTGCTTTCAC*C*A*G*T*G*AGATGT*C*G*T*G*A*C*G*T*GGAT TTTTCC*A*C*G*T*T*CTTTAATAGTGGACTCTTGTTCCAAACTGGAACA

**5-Dye**	Dye-TGTTCCAAATAGCCAAGCGGTCCACGCTCCCTGAGGGGCGCCAGGGTGGGAATCGGACAAGAGTCCACTAAAATCCCCCCAGCA
**6-Dye**	Dye-CATTAATTTTTTCTCCTTCACCGCCTGGGGTTTGCTTATAAATCAAAAGGTTTGGACCAACGCGCGGGGAGCGTATTAGAGTTG

* indicates a phosphorothioate (PPT) group between the two nucleosides.

2.2 Supplementary Figures

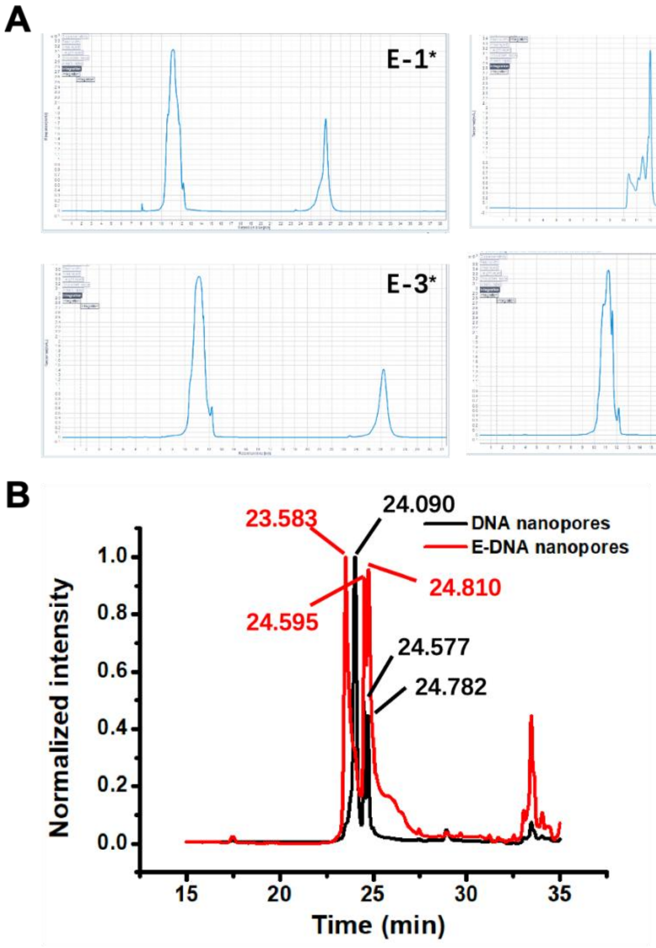

Supplementary Figure 1. (A) The HPLC results of E-1*, E-2*, E-3*, and E-4*. (B) The HPLC results of DNA nanopores and E-DNA nanopores.

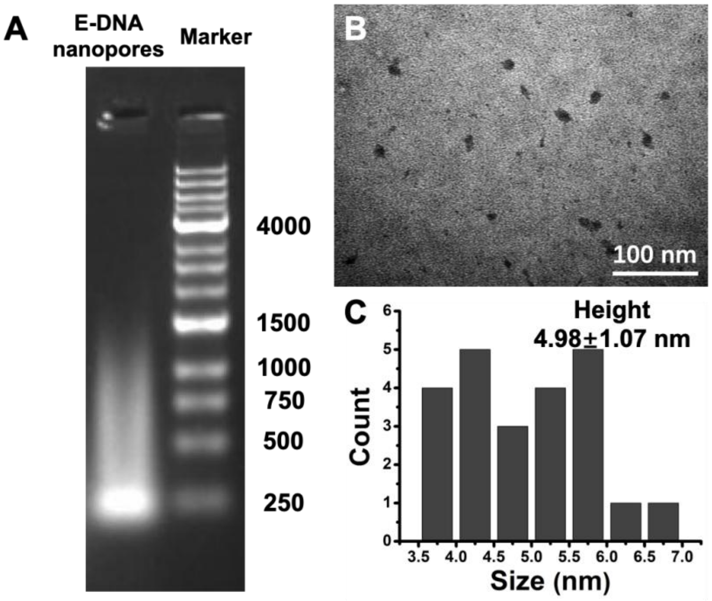

Supplementary Figure 2. Characterization of E-DNA nanopores. (A) Agarose gel electrophoresis analysis of E-DNA nanopores. Lane 1: E-DNA nanopores. Lane 2: 1kb DNA ladder. (B) TEM images of E-DNA nanopores. Scale bar: 100 nm. (C) Statistic results of the height of E-DNA nanopores according to AFM images (n=23).

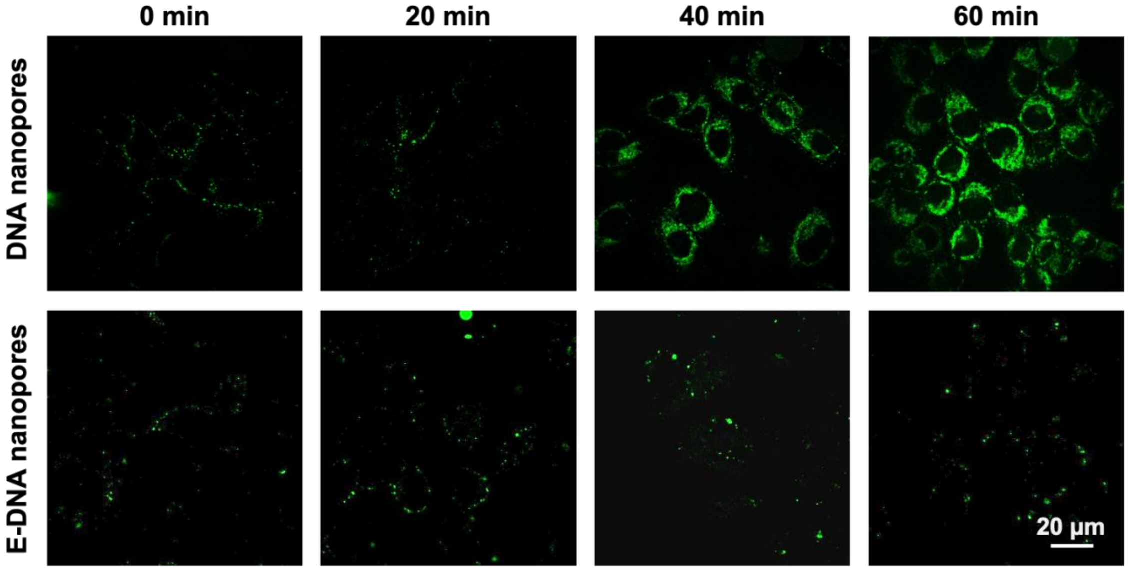

Supplementary Figure 3. Confocal images of MCF-7 cells incubated with DNA nanopores or E-DNA nanopores at 4°C for 40 min and then incubated at 37°C for different time. Scale bar: 20 μm.

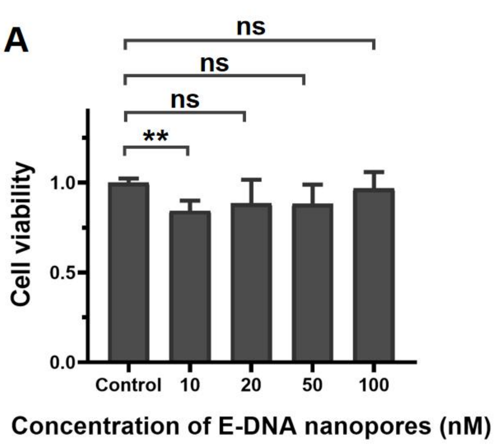

Supplementary Figure 4. CCK-8 assay results of MCF-7 cells incubated with different concentration of E-DNA nanopores for 2 h or incubated with 100 nM E-DNA nanopores for different time (n=5). ns indicates no significant difference. ** indicates P<0.01.

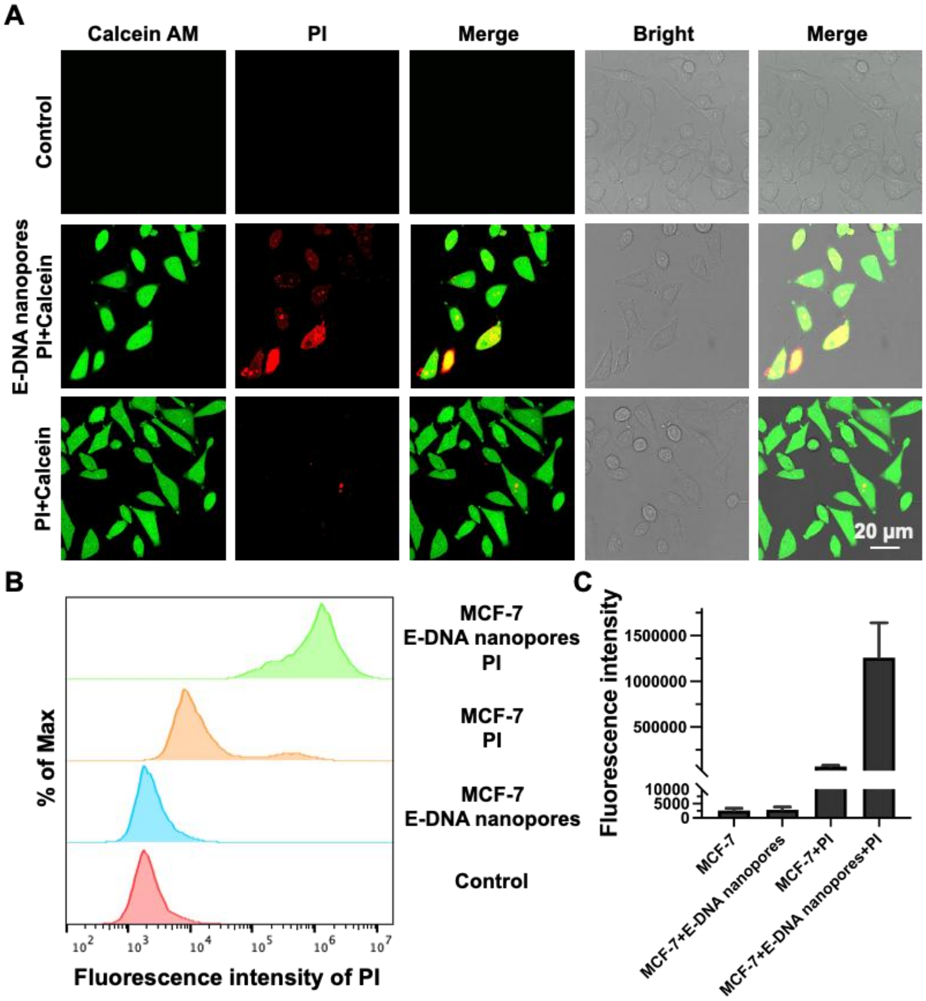

Supplementary Figure 5. (A-B) Fluorescence images and flow cytometry of MCF-7 cells inserted with E-DNA nanopores incubated with Calcein AM and PI. Scale bar: 20 μm. (C) The statistic results of PI fluorescence intensity according to flow cytometry.
